# Multi-loop atomic Sagnac interferometry

**DOI:** 10.1038/s41598-021-95334-7

**Published:** 2021-08-09

**Authors:** Christian Schubert, Sven Abend, Matthias Gersemann, Martina Gebbe, Dennis Schlippert, Peter Berg, Ernst M. Rasel

**Affiliations:** 1 Deutsches Zentrum für Luft- und Raumfahrt e.V. (DLR), Institut für Satellitengeodäsie und Inertialsensorik, c/o Leibniz Universität Hannover, DLR-SI, Callinstraße 36, 30167 Hannover, Germany; 2 Gottfried Wilhelm Leibniz Universität Hannover, Institut für Quantenoptik, Welfengarten 1, 30167 Hannover, Germany; 3 Zentrum für angewandte Raumfahrttechnologie und Mikrogravitation (ZARM), Universität Bremen, Am Fallturm, 28359 Bremen, Germany

**Keywords:** Matter waves and particle beams, Atomic and molecular interactions with photons, Ultracold gases

## Abstract

The sensitivity of light and matter-wave interferometers to rotations is based on the Sagnac effect and increases with the area enclosed by the interferometer. In the case of light, the latter can be enlarged by forming multiple fibre loops, whereas the equivalent for matter-wave interferometers remains an experimental challenge. We present a concept for a multi-loop atom interferometer with a scalable area formed by light pulses. Our method will offer sensitivities as high as $2\times 10^{-11}$ rad/s at 1 s in combination with the respective long-term stability as required for Earth rotation monitoring.

## Introduction

Rotation measurements are utilised for inertial navigation and earth observation exploiting the large enclosed area in fibre-optical and meter-scale ring laser gyroscopes^1–3^. Atom interferometry offers a different approach for providing absolute measurements of inertial forces with high long-term stability. Moreover, achieving the necessary areas for competitive performance with matter waves is a long-standing challenge^4–10^. We propose an atom interferometer performing multiple loops in free fall. Our setup opens the perspective for sensitivities as high as $2\times 10^{-11}$ rad/s at 1 s, comparable to the results of the ring laser gyroscope at the geodetic observatory Wettzell^1,2^.

The interferometric Sagnac phase shift^11^ induced by a rotation $\varvec{\Omega }$ depends linearly on the area vector $\varvec{A}$ as described in the following equation

1 $$\begin{aligned} \Delta \phi _{\mathrm {Sagnac}} = \frac{4\pi E}{\hbar c}\varvec{A}\varvec{\Omega } \end{aligned}$$

where *E* is the energy associated with the atom $E_{\mathrm {at}}=mc^2$ or photon $E_{\mathrm {ph}}=\hbar \omega$, *m* is the mass of the atom, $\omega$ the angular frequency of the light field, and *c* the speed of light. Since $E_{\mathrm {at}}\gg E_{\mathrm {ph}}$, it scales favourably for atoms, motivating early experiments^12–14^, while much larger areas were demonstrated for light^2^.

**Figure 1:**
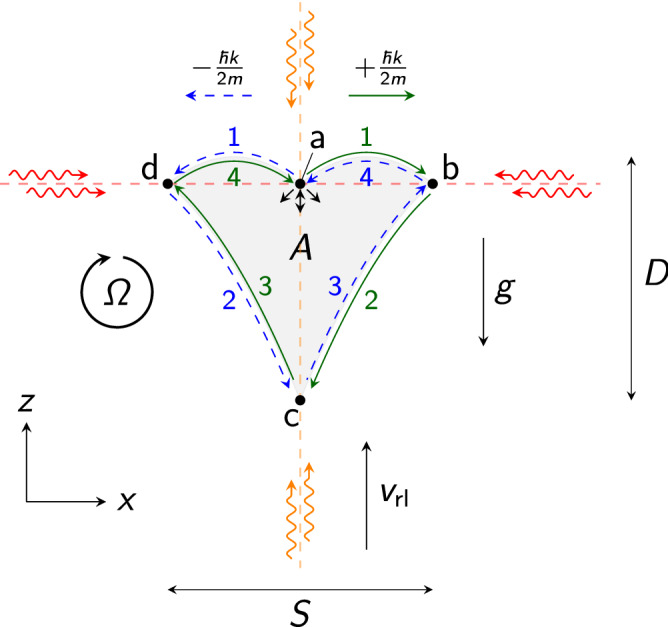
Trajectories of the free falling atoms in the interferometer. Red arrows denote the light fields for splitting, redirecting, and recombination. Orange arrows indicate the light fields for relaunching the atoms against gravity *g* with a velocity $v_{\mathrm {rl}}$ enabling operation with a single beam splitting axis (red) and closing the interferometer at its starting point. The atoms start at (a) where a beam splitting pulse leads to a coherent superposition of two momentum states (blue, green) that separate symmetrically with a recoil velocity of $\pm \hbar k /(2m)$. Here, *k* denotes the effective wave number of the beam splitter (red) and *m* the atomic mass. One momentum state follows the green arrows and the second one the dashed blue arrows according to the numbering. The state deflected in positive x-direction (green) propagates from (a) to (b), (c), (d), and back to (a). Similarly, but with inverted momentum, the other state (blue) proceeds from (a) to (d), (c), (b), and back to (a), closing the loop. As a consequence, the interferometer encloses the area *A* (grey shaded area), rendering it sensitive to rotations $\Omega$. Both trajectories meet at (c) where the two momentum states are relaunched at the same time. Input (up) and output ports (down) of the interferometer are indicated by black arrows below (a). The maximum wave packet separation is indicated by *S* and the drop distance by *D*.

One avenue for rotation measurements are wave guides or traps moving atoms in loops, especially for applications requiring compact setups^3^. Multiple loops were created in ring traps employing quantum degenerate gases for different purposes including the investigation of superconductive flows^15,16^. Exploiting them for guided atomic Sagnac interferometers in optical or magnetic traps remains an experimental challenge^17–20^.

**Figure 2:**
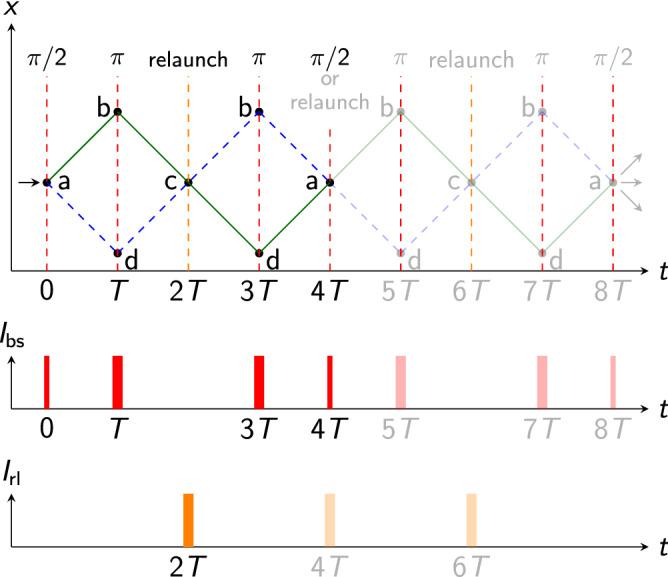
Space-time diagram and pulse timings of a multi-loop interferometer. The upper diagram shows the timing of the $\pi /2$ beam splitting at position (a) in Fig. 1, $\pi$ mirror pulses at position (b) and (d) in Fig. 1, and relaunch at position (c) in Fig. 1 as well as the recombination ($\pi /2$) pulse at (a). Non-opaque lines indicate an implementation with the minimum of two loops ($n=1$) and opaque lines a four-loop interferometer ($n=2$). The interferometer can be extended to 2*n* loops closed with a $\pi /2$ pulse at 4*nT* by introducing relaunches at $r\cdot 4T$ for $r\in (1,2,\ldots ,n-1)$ (position (a) in Fig. 1). The lower diagram shows the time-dependent intensities of the beam splitting pulses $I_{\mathrm {bs}}$ and relaunches $I_{\mathrm {rl}}$. Diagrams are not to scale, neglect pulse shaping and the initial launch before the atoms enter the interferometer.

Another approach is based on atoms in free fall. Here, light fields driving Raman or Bragg transitions coherently split, deflect, and recombine atomic wave packets in interferometers based on three^6,8^ or four pulses^5,7,9^, implementing a single loop or two loops, respectively. Operating a three-pulse interferometer with a thermal cesium atoms in a 2 m long vacuum chamber enabled the demonstration of a noise floor of $9\times 10^{-10}$ (rad/s)/$\sqrt{\mathrm {Hz}}$ and a long-term instability of $3\times 10^{-10}$ rad/s^10^. Aiming for more compact setups, sensors with a similar pulse configuration, but based on cold atoms with slow drift velocities showed sensitivities down to $10^{-7}$ rad/s in 1 s close to the shot noise limit^6,8^, motivating an increase of the enclosed area to enhance the sensitivity. Four-pulse interferometers^7,9^ enabled an increase of the enclosed area to up to $11\,\mathrm {cm}^2$, and reached sensitivities down to $3\times 10^{-8}$ rad/s in 1 s and $3\times 10^{-10}$ rad/s after averaging^5^. Dual or multi-loop interferometers have also been proposed in the context of terrestrial and space-borne infrasound gravitational wave detection designed for measuring strain rather than rotations^21–25^. Using molasses cooled atoms at microkelvin temperatures implies limits to the beam splitting efficiency^26,27^ and consequently to the contrast of the interferometer in the rotation sensor^5,6^. Additional cooling steps such as evaporative cooling provide a mitigation strategy^26^, thus enabling atom interferometers with high contrast^28–30^, higher-order beam splitters as a route for enhancing their sensitivity^30–41,41–44^, and efficient launch mechanisms^28,29^, which our concept builds on.

In our geometry (Fig. 1), atoms are coherently manipulated by two perpendicular light gratings (red and orange dashed lines) to form a multi-loop interferometer^23^. Pulsed light fields enable symmetric beam splitting of the atomic wave packets in the horizontal axis (red arrows)^41,43–45^ and relaunching in vertical direction (orange arrows)^28^. This approach offers a variety of advantages: (i) the free-fall time can be tuned to scale the area, (ii) the area is well defined by velocities imprinted during the coherent atom-light interactions, and (iii) the geometry utilises a single axis for beam splitting which avoids the requirement for relative alignment^5,23,46^. (iv) It enables multiple loops, (v) and its symmetry suppresses biases due to light shifts associated with the atom-light interaction. (vi) Moreover, our concept in principle allows incorporation of several additional measurements such as local gravity^47,48^ and tilt of the apparatus^43^ with respect to gravity.

## Multi-loop geometry

**Table 1:** Comparison of our multi-loop scheme with a four-pulse interferometer and performance estimation.

Sensor features	*N*	*k* $\left( \frac{2\pi }{780\,\mathrm {nm}} \right)$	*T* (ms)	*n*	*C*	*A* $\left( \mathrm {m}^2 \right)$	$t_{\mathrm {c}}$ (s)	*S* (m)	*D* (m)	Sensitivity $\left( \frac{\mathrm {rad}/\mathrm {s}}{\sqrt{\mathrm {Hz}}} \right)$
1: Multi loop	10^5^	**40**	10	10	1	4.6 × 10^−5^	1.6	**2.4** × **10**^−**3**^	**2.8** × **10**^−**3**^	3.2 × 10^−8^
1: Four pulse	10^5^	**350**	10	–	1	4 × 10^−5^	1.24	**2.1** × **10**^−**2**^	**5** × **10**^−**3**^	3.2 × 10^−8^
2: Multi loop	4 × 10^5^	20	250	10	1	**3.6** × **10**^−**1**^	**11.8**	3 × 10^−2^	0.7	**5.5** × **10**^−**12**^
2: Four pulse	4 × 10^5^	28	189	–	1	**2.1** × **10**^−**2**^	**2.8**	3.1 × 10^−2^	0.7	**4.2** × **10**^−**11**^
Compact	5.9 × 10^4^	40	10	6	0.53	2.8 × 10^−5^	1.44	2.4 × 10^−3^	2.8 × 10^−3^	1.2 × 10^−7^
High sensitivity	2.9 × 10^5^	20	250	4	0.66	1.4 × 10^−1^	5.8	3 × 10^−2^	0.7	1.7 × 10^−11^

We base our calculations on rubidium atoms^49^, a number of *N* detected atoms, an effective wave number *k*, a pulse separation time *T* (see Fig. 2), and a contrast *C*, performing 2*n* loops. We denote *A* as the effectively enclosed area, which scales with *n*. Both the calculation of *A* and the sensitivity neglect finite pulse durations. The maximum trajectory separation is given by *S*. For the estimation of the drop distance *D* with regard to a compact scenario we allow for additional time of 6 ms for the momentum transfer. Our interferometer cycle time is denoted by $t_{\mathrm {c}}$ and we state the sensitivity in the shot-noise limit according to Eq. (4). In the first four rows we compare calculated parameters of multi-loop interferometers to four-pulse geometries without a relaunch^5,7,9^, emphasising differences in the parameters in bold, and neglecting atom losses and contrast reduction due to imperfect beam splitters. For the lower two rows, we assume a simple model in which the contrast for multiple loops *C*(*n*) decreases depending on the number of loops *n* scaling as $C(n)=C(1)^{n}$ with *C*(1) denoting the contrast for a single loop. Furthermore, our model reduces the number of detected atoms by a factor $l^{n-1}$ with $l=0.9$ for 2*n* loops to take inefficiencies in the atom-light interactions into account.

Conceptually, our idea exploits multiple loops in the interferometer to effectively increase the enclosed area. When neglecting losses of contrast and atoms which may scale with the number of loops due to imperfections in the atom-light interactions, this implies a linear increase in the shot-noise limit of the sensitivity per cycle. The concept of our interferometer is detailed in Fig. 1, showing the two trajectories (blue, green) that enclose an area $\varvec{A}$. We now describe the sequence to implement our scheme. Initially, an atomic wave packet is launched vertically. On its upward way, the wave packet interacts with the horizontal beam splitter with an effective wave number *k* forming two wave packets drifting apart with a momentum of $\pm \hbar k/(2m)$ (a). After a time *T*, the horizontally oriented light field (red) inverts the movement of the atoms on its axis (b,d). On their way downwards due to gravity, the vertically oriented light field (orange) relaunches the atoms^28^ at the lowest point of the interferometer at 2*T* (c) reversing their momentum to move upward. The atoms pass the horizontal atom-light interaction zone again at 3*T* (b,d) where they are deflected towards each other and cross falling downwards at 4*T* (a) completing a double loop. In order to start the next loop, they are relaunched (see Fig. 2). Repetition of the procedure determines the number of loops 2*n*. After the last loop, the interferometer is closed by flashing a beam splitter pulse instead of an upward acceleration. Due to the specific implementation of the initial launch at position (a) and relaunch at (c), our concept only requires a single beam splitting zone (red) for the splitting and deflection operations at (a), (b) and (d)^23^ instead of two^5,7,9^ or three^6,8,10^.

The acquired Sagnac phase shift depends on the total time of the interferometer 4*T*, the effective wave vector $\varvec{k}$, local gravity $\varvec{g}$, number of loops 2*n* and reads

2 $$\begin{aligned} \Delta \phi _{\mathrm {Sagnac}} = n\cdot 4(\varvec{k}\times \varvec{g})\varvec{\Omega } T^3\mathrm {{,}} \end{aligned}$$

calculated with the methods outlined in ref.^50–52^ and similar as in refs.^5,7,9,53^. The relaunch velocity $v_{\mathrm {rl}}=|\varvec{v}_{\mathrm {rl}}|=3gT$ with $g=|\varvec{g}|$ is aligned parallel to gravity and is chosen to close the atom interferometer at its starting point (position (a) in Fig. 2). In this configuration, the effectively enclosed area is given by

3 $$\begin{aligned} A = n\cdot 2\frac{\hbar k}{m}g T^3. \end{aligned}$$

It can be enlarged by a higher transverse momentum $\hbar k=\hbar |\varvec{k}|$, e.g. by transferring more photon recoils, and by increasing the free fall time 4*T* of the interferometer.

Enlarging the number of loops by a factor *n* effectively increases the enclosed area without changing the dimensions of the geometry. We define them as the maximum wave packet separation in the horizontal axis $S=\hbar k T/m$ and the drop distance in the vertical axis $D=(3T/2)^2\cdot g/2$. The relaunch at 4*T* (or multiples of 4*T* for more than four loops, position (a) in Fig. 1) reuses the same light field (orange arrows) as for the first relaunch at 2*T* (position (c) in Fig. 1) and does thus not add complexity.

To estimate the potential sensitivity of a future experiment, we calculate the shot-noise limit based on the phase shift in Eq. (2) including the dependency on the finite cycle time. Typically, the cycle of an atom interferometer consists of the generation and preparation of the atomic ensembles during the time $t_{\mathrm {prep}}$, the interferometer time which for our geometry reads $n\cdot 4T$, and detecting the population of the output ports within the time $t_{\mathrm {det}}$. This leads to a total cycle time of $t_c=t_{\mathrm {prep}}+n\cdot 4T+t_{\mathrm {det}}$. For *N* detected atoms and an interferometer contrast *C* of the interferometer, the shot-noise limited sensitivity to rotations $\Omega _{y}$ is given by

4 $$\begin{aligned} \sigma _{\Omega }(t) = \frac{1}{C\sqrt{N}\cdot n\cdot (4kgT^3)}\sqrt{\frac{t_{\mathrm {prep}}+n\cdot 4T+t_{\mathrm {det}}}{t}} \end{aligned}$$

after an averaging time *t* corresponding to multiples of the cycle time $t_{\mathrm {c}}$. Consequently, an interferometer with a small free fall time $4T\ll t_{\mathrm {prep}}+t_{\mathrm {det}}$ benefits more from multiple loops with a scaling of $\sim 1/n$ in Eq. (4) than other scenarios with $4T\thickapprox t_{\mathrm {prep}}+t_{\mathrm {det}}$ that scale as $\sim 1/\sqrt{n}$. Implementing an interferometer time $n\cdot 4T > t_{\mathrm {prep}}$ can enable a continuous scheme by sharing $\pi /2$ pulse between subsequent interferometers^5,54^. In general, our geometry offers the possibility to compensate smaller *T* with an appropriate *n*.

Table 1 (row 1, 3) reports the calculated shot-noise limited sensitivities according to Eq. (4) for two different implementations of our geometry.

## Spurious phase shifts

Spurious phase shifts may degrade the sensitivity of the sensor if they are not inherently suppressed or sufficiently well controlled. The choice of symmetric beam splitting in our geometry suppresses phase noise of the beam splitting lasers, as well as the impact of spatially homogeneous AC-Stark and magnetic field shifts on the two arms of the interferometer^43,44^. We refer to dedicated studies for impacts of light shifts due to large momentum beam splitters^34,41,55–59^, and isolation or correlation methods to remove vibration noise^47,60–65^ for quantum sensors which we expect to be exploited in a future experimental realisation of our concept.

In multi-loop interferometers, the sensitivity to DC accelerations and phase errors depending on the initial position and velocity is suppressed when compared to a three-pulse or single-loop atom interferometer^25,53^. Still, spurious couplings remain which we now assess following the methods of refs.^50–52^. We start with a discussion of phase shifts introduced by the relaunch, and then continue with phase shifts that depend on the starting position or velocity, and the gravity gradient, that may constrain these parameters. Here, we only focus on terms we estimate to be dominating.

A non-ideal pointing of the relaunch velocity $\varvec{v}_{\mathrm {rl}}$ may introduce spurious phase shifts in a real setup. We consider small deviations $\alpha =|\varvec{v}_{\mathrm {rl}}\times \varvec{e}_{x}|/(|\varvec{v}_{\mathrm {rl}}||\varvec{e}_{x}|)$ and $\beta =|\varvec{v}_{\mathrm {rl}}\times \varvec{e}_{y}|/(|\varvec{v}_{\mathrm {rl}}||\varvec{e}_{y}|)$ in a double-loop configuration. Here, $\varvec{e}_{x}=\varvec{k}/|\varvec{k}|$ denotes the unit vector in x-direction and $\varvec{e}_{y}=(\varvec{k}\times \varvec{g})/(|\varvec{k}||\varvec{g}|)$ denotes the unit vector in y-direction (see Fig. 1).

If the timing of the relaunch is not ideally centred around 2*T*, but shifted by $\delta \tau$, coupling to non-zero $\alpha$ leads to the phase shift^23^

5 $$\begin{aligned} \Delta \phi _{\alpha ,\tau }=-kv_{\mathrm {rl}}\alpha \delta \tau =-3kgT\alpha \delta \tau . \end{aligned}$$

Provided the pointing of the relaunch velocity is adjustable (e.g. with a tip-tilt mirror controlling the alignment of the light field for relaunching) $\alpha$ and $\delta \tau$ can be adjusted by iteratively scanning both.

In addition, tilting the relaunch vector induces phase shifts resembling those of a three-pulse or Mach-Zehnder-like interferometer^50,51^ by coupling to gravity gradients $\Gamma$ and rotations $\varvec{\Omega }$. These contributions read

6 $$\begin{aligned} \Delta \phi _{\alpha ,\Gamma } = \varvec{k}\Gamma \varvec{v}_{\mathrm {rl}}T^3 = 3k\alpha \Gamma _{x} gT^4, \end{aligned}$$

with $\Gamma _i=\varvec{e}_{i}\Gamma \varvec{e}_{i}$ for $i=x,y,z$ and

7 $$\begin{aligned} \Delta \phi _{\beta ,\Omega } = 2 \left( \varvec{k}\times \varvec{v}_{\mathrm {rl}} \right) \cdot \varvec{\Omega }T^2 = 6k\beta g\Omega _{z}T^3, \end{aligned}$$

corresponding to a spurious sensitivity to a rotation $\Omega _{i}=\varvec{e}_i\cdot \varvec{\Omega }$ for $i=x,y,z$.

Scanning the interferometer time 4*T* enables an iterative procedure to minimise spurious phase shifts by optimising the contrast^46^.

We now show the phase terms stemming from a coupling of starting position $\varvec{r}_0=(x_0,y_0,z_0)$ and velocity $\varvec{v}_0=(v_{x0},v_{y0},v_{z0})$ to rotations and gravity gradients, as well as the cross coupling between the latter. According to our estimation (see Table 1 for our choice of parameters for *k*, *T*, but using $n=1$), the dominating terms read:

8 $$\begin{aligned} \Delta \phi _{vx}= & {} 4k T^3 \left( \Gamma _x + 3 \left( \Omega _y^2 + \Omega _z^2 \right) \right) v_{x}, \end{aligned}$$

9 $$\begin{aligned} \Delta \phi _{vy}= & {} -4k T^3 \left( 3 \Omega _x \Omega _y +4 T \left( \Gamma _x + \Gamma _y \right) \Omega _z \right) v_{y}, \end{aligned}$$

10 $$\begin{aligned} \Delta \phi _{vz}= & {} -4k T^3 \left( 3 \Omega _x \Omega _z + 4 T \left( \Gamma _z + \Gamma _x \right) \Omega _y \right) v_{z}, \end{aligned}$$

11 $$\begin{aligned} \Delta \phi _{y0}= & {} 8k T^3 \Gamma _y \Omega _z y_0, \end{aligned}$$

12 $$\begin{aligned} \Delta \phi _{z0}= & {} -8k T^3 \Gamma _z \Omega _y z_0, \end{aligned}$$

13 $$\begin{aligned} \Delta \phi _{\Gamma x}= & {} 18 k T^5 \Omega _y \Gamma _x,\,\,\mathrm {and} \end{aligned}$$

14 $$\begin{aligned} \Delta \phi _{\Gamma z}= & {} 18 k T^5 \Omega _y \Gamma _z. \end{aligned}$$

Phase terms scaling as $\Delta \phi _{\Gamma y} \sim k T^6 g \Gamma _y \Omega _x \Omega _z$ and $\Delta \phi _{x0} \sim k T^4 \Gamma _x^2 x_0$ that may constrain $\Gamma _y$ and $x_0$ are estimated to be negligible for our choice of parameters. Optionally, a compensation scheme implemented by small frequency detuning of the $\pi$ pulses^21,66–68^ at $(r\cdot 4-3)T$ and $(r\cdot 4-1)T$ for $r\in (1,2,\ldots ,n-1)$ in Fig. 2 (b, d) can reduce the impact of the gravity gradient $\Gamma _x$ in Eq. (9). Additionally, we have to consider constraints from the beam splitting process. The velocity acceptance of the beam splitter^41,43^ may limit $v_x$. Another constraint is to have the atoms within the central part of the beam splitter both at the start, limiting $y_0$ and $z_0$, and at the last pulse for recombination, limiting $v_y$ and $v_z$. The impact of a variation in *g* (see Eq. 2) may be reduced with a tidal model similar to the common practice in gravimeters^47,61,65,69^.

Table 2 reports the calculated requirements for two different implementations of our geometry.

## Perspectives of the performance

Apart from the (re-)launch, our scheme with $n=1$ resembles four-pulse interferometers^5,7,9,53^ in geometry and scale factor (Eqs.  2 and 3 ). Hence, we show the advantages of our method with respect to a four-pulse interferometer for two design choices. For both interferometers in Table 1 (upper four rows), we assume ideal contrast, no losses of atoms, as well as shot-noise limited sensitivities (see Eq.  4). (1) Matching the free-fall time *T* and sensitivity for the multi-loop and four-pulse sensor, the four-pulse interferometer requires a larger photon momentum transfer in horizontal direction. Consequently, the size $S\cdot {D}$ of the multi-loop geometry is by a factor of 15 smaller (emphasised in bold in Table 1). (2) Aiming for similar dimensions *S* and *D*, we obtain a nearly an order of magnitude higher sensitivity for our multi-loop geometry at the cost of an increased cycle time.

**Table 2:** Requirements on the pointing of the relaunch, starting parameters, variation of gravitational acceleration, and its gradient.

	$\alpha _{\delta \tau } (rad)$	$\alpha _{\Gamma }$	$\beta$	$v_{x0}$ ($\upmu$m/s)	$v_{y0}$	$v_{z0}$	$y_0$ ($\upmu$m)	$z_0$	${\delta }g$ (m/s$^2$)	${\delta }\Gamma$ (1/s$^2$)
Compact	$1.3\,\times 10^{-4}$	$<0.1$	$9.4\,\times 10^{-5}$	200 $^{*}$	250 $^{\dagger }$	250 $^{\dagger }$	100 $^{\ddagger }$	100 $^{\ddagger }$	$5.6\,\times 10^{-4}$	$7.2\times 10^{-2}$
High sensitivity	$6\,\times 10^{-6}$	$2.5\,\times 10^{-6}$	$6.6\,\times 10^{-9}$	26 $^{\star }$	10 $^{\dagger }$	10 $^{\dagger }$	100 $^{\ddagger }$	100 $^{\ddagger }$	$5.4\,\times 10^{-8}$	$1.1\times 10^{-8}$

The parameters are calculated to induce contributions (see Eqs.  5 to 14) by a factor of $10\cdot {n}$ below the shot-noise limit ($1/\sqrt{N}$) for the scenarios in the lower rows of Table 1. We assume $\delta \tau =10\,\mathrm {ns}$ for a typical experiment control system^31^, Earth’s gravity gradient $\Gamma _{x}=\Gamma _{y}=-0.5\Gamma _{z}=1.5\times 10^{-6}\,\mathrm {s}^{-2}$, and $\Omega _{x}=\Omega _{y}=\Omega _{z}=7.27\times 10^{-5}$ rad/s using Earth’s rotation rate as an upper limit^50,51^.

$^*$Limited by the velocity acceptance of the beam splitter^41,43^.

$^\star$Assuming a gravity gradient compensation^21,66–68^ to $0.1\cdot \Gamma _{x}$, neglecting the impact on other terms as the small change in the enclosed area^66^.

$^\dagger$Requirement set to limit the change in position w.r.t. the beam splitter between first and last pulse to $100\,\upmu \mathrm {m}$.

$^\ddagger$Constraint to have the atoms within $100\,\upmu \mathrm {m}$ of the center of the beam splitter at the first pulse.

Showcasing more realistic scenarios, we consider a simple model for losses of atoms and reduction of contrast in dependence on the number of loops as summarised in Table 1 (lower rows). The latter can result from inhomogeneities of the light fields^34,41,55–59^. Following our calculations and choice of parameters, we still anticipate a compact sensor with a sensitivity of $1.2\times 10^{-7}\,(\mathrm {rad}/\mathrm {s})/\sqrt{\mathrm {Hz}}$ within a volume of 20 mm$^3$ for the interferometer, and a highly sensitive, but larger device with $1.7\times 10^{-11}\,(\mathrm {rad}/\mathrm {s})/\sqrt{\mathrm {Hz}}$ within a meter-sized vacuum vessel based on our method, comparable to the performance of large ring laser gyroscopes^2^.

Multiple experiments investigated beam splitting as well as relaunch operations as required for our scheme. They realised the transfer of large momenta with subsequent pulses or higher order transitions^32–37^ and their combination with Bloch oscillations^30,38,39^. The implementation of symmetric splitting^40–44^ was demonstrated with an effective wave number corresponding to 408 photon recoils in a twin-lattice atom interferometer^41^. A similar procedure enabled the relaunch of atoms^28^. The requirement for high efficiency implies using atomic ensembles with very low residual expansion rates^26^ as enabled by delta-kick collimation of evaporated atoms^31,70^ and Bose-Einstein condensates^28,29,41,71,72^. In addition, interferometers exploiting such ensembles may benefit from the suppression systematic of uncertainties^73–76^. Fountain geometries utilised launch techniques compatible with these ensembles^28,29,31,70^. Rapid generation of Bose-Einstein condensates with $10^5$ atoms was demonstrated^77,78^ and realised with atom chips in 1 s^79,80^ which we adopted for our estimation.

Reaching the shot-noise limited sensitivity implies a restriction on tilt instability as detailed in Table 2 due to couplings in Eqs.  5, 6, and 7. It is approximately met at the modest level of $0.1\,\mathrm {mrad/}\sqrt{\mathrm {Hz}}$ for the compact scenario and at $7\,\mathrm {nrad/}\sqrt{\mathrm {Hz}}$ for high-sensitivity. Dedicated vibration isolation systems demonstrated a noise floor of $1\,\mathrm {nrad/}\sqrt{\mathrm {Hz}}$ in a frequency range of 1 Hz to 100 Hz^81^. Alternatively, and similar as in a large ring laser gyroscope^1,2^, tiltmeters with a resolution of sub nrad^82^ may enable post correction methods. The requirements on the stability of the starting position and velocity appear to be within reach of current sources of Bose-Einstein condensates^28,71,73,74,80,83^.

## Conclusion and discussion

We presented our concept for an atomic gyroscope capable of performing multiple loops by exploiting light pulses for beam splitting and relaunching atoms with the perspective of reaching unprecedented sensitivities for rotations. It offers unique scalability in a sensor head with a limited size. Key elements as the symmetric beam splitting^41,43^, relaunch^28^, as well preparation of the ultracold atoms^70,72,79,80,84^ have already been demonstrated. The tools for coherent manipulation in our scheme additionally allow for the implementation of geometries for a tiltmeter^43^ and a gravimeter^28,47,48^. We showed the perspective for compact setups, which can be scaled up to compete with large ring laser gyroscopes^1,2^. This might enable the detection of multiple rotational components in a single set-up by adding a second orthogonal beam splitting axis, and sensitivities as required for measuring the Lense-Thirring effect^85–88^.

## Data Availability

Datasets are available on reasonable request.
